# Need to know: the need for cognitive closure impacts the clinical practice of obstetrician/gynecologists

**DOI:** 10.1186/s12911-014-0122-6

**Published:** 2014-12-24

**Authors:** Greta B Raglan, Maxim Babush, Victoria A Farrow, Arie W Kruglanski, Jay Schulkin

**Affiliations:** Department of Research, American College of Obstetricians and Gynecologists, 409 12th St, SW, Washington, DC 20024 USA; Department of Psychology, American University, Washington, DC 20016 USA; Department of Psychology, University of Maryland, College Park, MD 20742 USA

**Keywords:** Need for Cognitive Closure, Obstetricians/gynecologists, Medical decision making, Uncertainty

## Abstract

**Background:**

Need for cognitive closure (NFCC) has been shown to be a consistent and measurable trait. It has effects on decision making and has been associated with more rapid decision making, higher reliance on heuristics or biases for decision making, reduced tolerance for ambiguity, and reduced interest in searching for alternatives. In medical practice, these tendencies may lead to lower quality of decision making.

**Methods:**

This study measured NFCC in 312 obstetrician/gynecologists using a survey-style approach. Physicians were administered a short NFCC scale and asked questions about their clinical practice.

**Results:**

Obstetrician/gynecologists with high NFCC were found to be less likely to address a number of clinical questions during well-woman exams, and were more likely to consult a greater number of sources when prescribing new medications.

**Conclusions:**

NFCC of physicians may have an important impact on practice. It is possible that increased training during residency or medical school could counteract the detrimental effects of NFCC, and steps can be taken through increased use of electronic reminder systems could orient physicians to the appropriate questions to ask patients.

**Electronic supplementary material:**

The online version of this article (doi:10.1186/s12911-014-0122-6) contains supplementary material, which is available to authorized users.

## Background

Accurate decision making is crucial in medicine, as mistakes can have dangerous consequences. Physicians, in spite of their extensive education and years of experience, are not immune to the impact of a low tolerance for ambiguity and uncertainty on judgment and decision-making. Previous studies have shown that physicians report intolerance of uncertainty similar to non-physicians [1]. In fact, some studies have indicated that medical practitioners, at least those in training, may have lower tolerance for uncertainty than non-physician populations [2]. Some studies have examined ambiguity tolerance in physicians and have found that it has an impact on clinical practice. For example, a study conducted by Carney and colleagues (2007) found that physicians with low tolerance of uncertainty were more likely to recall patients for additional testing following mammograms, possibly leading to unnecessary, costly, and distressing procedures [3]. Others have found that physicians with low tolerance of uncertainty were more likely to make recommendations on terminating pregnancy after prenatal genetic testing and were also more likely to withhold negative test results [4].

One group of people that is particularly averse to ambiguity and uncertainty is those with a high need for cognitive closure. Need for cognitive closure (NFCC) is a “desire for definite knowledge on some issue [5]”. NFCC has been shown to be a consistent and measurable trait among individuals such that some individuals have a generally higher or lower NFCC [6]. High NFCC has been associated with more rapid decision making, higher reliance on heuristics or biases for decision making, reduced tolerance for ambiguity, and reduced interest in searching for alternatives [7-9]. NFCC is defined by individuals’ tendency to “seize” onto specific information, and to “freeze” their response to that information and avoid conflicting ideas [5,8]. Previous studies have shown that in medical trainees, those with less experience tend to be more influenced by NFCC, such that they are more likely to “freeze” when making diagnostic decisions, but that the effect of NFCC declines with experience [10]. NFCC is thought to allow the individual greater perceived certainty, but in some contexts it may restrict the information available to the person and could lead to greater chances of poor decision making.

These tendencies may be particularly strong in obstetricians [4] and, given that NFCC could influence the judgments and decisions of medical practitioners [10], it is important to better understand precisely how physicians’ NFCC and intolerance of uncertainty might affect their clinical decision making and their use of information sources when making decisions. The purpose of this study was to examine NFCC in obstetrician/gynecologists (ob/gyns), and relate it to ob/gyns’ socio demographic characteristics and practice patterns. We hypothesized that ob/gyns with high NFCC would query fewer sources when deciding to prescribe a new drug, would consult with fewer specialist providers, would be less likely to ask screening questions about a variety of conditions in well-woman visits, and would be less likely to perform trial of labor or vaginal birth after cesarean (TOLAC/VBAC) under conditions of increased risk.

## Methods

Six-hundred members of the American College of Obstetricians and Gynecologists’ Collaborative Ambulatory Research Network (CARN), a group of over 2,000 fellows who voluntarily participate in four to six surveys annually without compensation, were sent a mailing including a cover letter, survey, and prepaid return envelope. Those who did not respond were sent three reminder mailings. After four total mailings were administered, fellows were considered nonresponders. This study received approval from the Institutional Review Board at Indiana University in August 2013. The funding source had no role in the current study.

Participants completed a 6-page questionnaire with 51-items, which covered a range of topics including: decision-making under uncertainty, consultation and preferred interventions for periviable patients, work experiences, and demographics. Items pertaining to NFCC included questions about resources used when prescribing new medications, frequency of consultation with specialists, the scope of well-woman exams, and conditions under which TOLAC/VBACs are performed. Responses were on Likert scales (e.g., 1 = Almost Never, 5 = Almost Always), or select all answers that apply. In addition, we used a shortened version of the Need for Closure Scale asking to what extent participants agreed with two dummy statements and 14 target statements (e.g., “I get very upset when things around me aren’t in their place”) on a scale of 1–6 (i.e., 1 = Strongly Disagree, 6 = Strongly Agree) [6]. This scale was scored by totaling the responses to the 14 target statements. Higher scores indicate higher need for closure.

Sociodemographic characteristics included: age, sex, race, medical specialty/subspecialty, number of years out of residency, practice setting, and location. Practice state was also collected to enable us to assess geographic practice variation. Surveys items were pretested on ob/gyn physicians at Indiana University Hospital. For additional details regarding the survey, please see Additional file 1.

The data were analyzed using a personal computer-based software package (IBM SPSS Statistics® 20.0, IBM Corp©, Armonk, NY). Descriptive statistics were computed for the measures used in the analyses. Group differences in responses on continuous measures were assessed with ANOVA analyses and linear regressions. Group differences on categorical measures were assessed with *χ*2 tests. Pearson’s correlations were used to describe correlations between continuous variables.

## Results

Three-hundred-twelve surveys were returned for a total response rate of 53.1%. Twelve participants were excluded because they could not be contacted or because they had retired from practice. Of the 312 participants who returned a survey, 252 (80.8%) completed the NFCC Scale, 58 (18.6%) did not complete the scale, and three (.01%) were excluded because they did not complete the survey. Respondents did not differ significantly from non-respondents in terms of sex (*χ*^*2*^ = .783, *p* = .376), age (*F* (1, 308) = .238, *p* = .595), or years post-residency (*F* (1, 300) = 1.573, *p* = .211). The mean score on the NFCC scale was 39.9 (min = 14; max = 66; SD = 9.75), and there were no differences between those who completed the NFCC scale and those who did not in terms of age, race, sex, years in practice, or practice characteristics (see Table 1). Further analyses include only those participants who completed the NFCC scale. No effects of demographic characteristics (e.g., age, race, sex) were found in NFCC, and the trait was normally distributed among participants.

**Table 1:** **Participant demographics**

	**Physicians completed scale (n = 252)**	**Physicians did not complete scale (n = 57)**	**Significance (** ***p*** **=)**
Age (mean years, SD)	53.8, 8.82	54.5, 9.91	.595
Sex (% Female)	50.6%	57.1%	.376
Race*			
White/European American	89.5%	85.5%	.388
Black/African American	4.0%	5.5%	.638
Hispanic/Latin American	3.2%	0.0%	.177
Asian/Pacific Islander	4.0%	7.3%	.300
Clinical Practice Setting			.055
Solo/Private Practice	12.9%	26.8%	
Partnership/Group Practice	45.2%	41.1%	
Multi-specialty Group	17.3%	8.9%	
University full time	12.1%	16.1%	
Other	7.1%	12.5%	
Practice Location			.942
Urban, inner city	18.2%	19.3%	
Urban, non-inner city	30.4%	30.4%	
Suburban	32.8%	30.4%	
Town of 5,000–50,000	15.4%	17.9%	
Rural/Other	3.2%	1.8%	

*Numbers may not equal to 100% due to individuals identifying with multiple categories.

The shortened NFCC scale showed high internal consistency (Cronbach’s α = .795). As hypothesized, higher NFCC scores were associated with less frequently screening patients for alcohol consumption *F*(1,230) = 8.77, *β* = -.021, *p* = .003, cigarette smoking *F*(1,229) = 9.38, *β* = -.014, *p* = .002, illegal drug use *F*(1,228) = 4.16, *β* = -.016, *p* = .042, prescription drug use *F*(1,230) = 6.89, *β* = -.018, *p* = .009, over the counter drug use *F*(1,230) = 10.5, *β* = -.023, *p* = .001, environmental toxins *F*(1,230) = 10.3, *β* = -.021, *p* = .002, sexual abuse *F*(1,229) = 8.83, *β* = -.022, *p* = .003, domestic violence *F*(1,230) = 11.1, *β* = -.024, *p* = .001, and mental health, *F*(1,230) = 6.89, *β* = -.016, *p* = .009, during periodic well-woman exams (WWEs). Addressing folic acid was marginally significant, *F*(1,229) = 3.56, *β* = -.012, *p* = .06. Addressing obesity (*p* = .17), exercise (*p* = .10), health history (*p* = .21), and caffeine use (*p* = .26), were not associated with NFCC. Additionally, NFCC was not associated with total number of health habits addressed during WWEs (*p* = .90). For additional information, see Table 2.

**Table 2:** **Relationships between need for closure and frequency of screening for preventative behaviors**

**Non-significant relationship**	**Significant relationship**	
Folic Acid	Alcohol Consumption	(-)
Obesity	Cigarette Smoking	(-)
Exercise	Illegal Drug Use	(-)
Health History	Prescription Drug Use	(-)
Caffeine	Over the Counter Drug Use	(-)
	Environmental Toxins	(-)
	Sexual Abuse	(-)
	Domestic Violence	(-)
	Mental Health	(-)

Contrary to initial expectations, high NFCC was associated with consulting a *greater* total number of *sources* when deciding to prescribe a new drug (*F*(1,251) = 4.06, *β* = .007, *p* = .045). Some relationships were observed between NFCC and the likelihood of consulting other physicians. Specifically, high NFCC predicted less frequent consultations with genetics counselors, *F*(1,247) = 4.43, *β* = -.019, *p* = .036, and neonatologists, *F*(1,245) = 6.44, *β* = -.023, *p* = .012,, but not with rates of consultation with other specialists. However, the relationship between NFCC and the overall frequency of specialist consultation was approaching significance, *F*(1,248) = 2.97, *β* = -.009, *p* = .086. For additional information, see Table 3.

**Table 3:** **Relationship between need for closure and likelihood of consulting specialist sources**

**Non-significant relationship**	**Significant relationship**	
**Overall frequency of specialist consultation**	**Total specialist sources consulted**	(+)
MFM Specialist	Genetic Counselors	(-)
Gynecological Oncologist	Neonatologists	(-)
Reproductive Endocrincologist		
Internist		
Other		

People high on NFCC were more likely to recommend referrals for non-pregnant patients presenting with a variety of disorders (*β* = .007, *t*(251) = 1.979, *p* = .054), but this relationship was marginally significant. There was no relationship between NFCC and the number of treatments considered, *p* = .469.

High NFCC was also associated with a lower likelihood of offering a TOLAC/VBAC to a patient with one prior low transverse C-section during the induction of labor, *F*(1, 214) = 6.40, *β* = -.023, *p* = .012. NFCC was not associated with the likelihood of offering a TOLAC/VBAC in other circumstances of labor (e.g., no prior deliveries, spontaneous labor, etc.).

## Discussion

Based on these findings, it appears that some of ob-gyns’ clinical practices are correlated with NFCC. We did not find that socio demographic characteristics such as sex, age, or years in practice since residency were associated with NFCC. Rather, we found that NFCC was a normally distributed trait among physicians regardless of age, sex, race, or clinical practice characteristics. We found that physicians with high NFCC refer to more information sources when prescribing a new drug. It is possible that high NFCC individuals are uncomfortable with change and innovation [11], and that is why they resist prescribing new drugs and tend to consult more sources than their low NFCC counterparts before doing so. Additionally, we found that higher NFCC is associated with reduced frequency of asking screening questions about certain conditions during WWEs. Overall, this study finds that NFCC is tied to less seeking out of medical conditions for which treatment may be required (i.e., fewer screening questions in WWEs, fewer consultations), but that more information is sought by high NFCC physicians in certain circumstances (i.e., when prescribing a new drug).

It is possible that where high NFCC practitioners feel expert, they tend to seek less information than their low NFCC counterparts, but when it comes to change and innovation, they are more conservative [12], hence they seek more information because of the discomfort of introducing change into their practices. Additionally, we found that ob-gyns with higher NFCC tended to consult genetics counselors and neonatologists less frequently. This may be because high NFCC persons, known to be authority-driven [12,13], could view some medical specializations as less knowledgeable than others and hence consult less with those medical specialties. Further research into ob/gyns’ perceptions of subspecialties may provide more information about this finding.

While this study has important findings, it also has limitations. Results shows moderate effect sizes for the phenomena of interest. Further studies are necessary to determine whether these effects can be replicated in larger samples. Additionally, this study relies on respondent self-reports and recall, which may be open to recall or response bias. Because this study is descriptive in nature, it is not possible to draw conclusions about causal relationships between groups. We did not find an effect of years in practice on NFCC. It is possible that participants in the current study are sufficiently experienced that NFCC impacts them differently than it would physicians earlier in their careers [10]. Studies looking into multiple physician groups, as well as physicians at different points in their training, may shed light on whether the observed findings are unique to our population, or if they are also present among other types of physicians and ob-gyns at different levels of training.

Preventive care and screening questions are important to providing quality care to patients, and prompted self-report can provide physicians with patient information for further examination. Appropriate preventive care and early interventions can reduce risks for many conditions and can improve outcomes. While the current study examined ob-gyns’ likelihood of asking about some conditions during a WWE, further research should be conducted into screening questions for other visits, and questions regarding other conditions or areas of intervention.

Some have advocated for incorporating measures of ambiguity tolerance or NFCC into admissions exams for medical schools, in an effort to increase this trait among physicians [14]. Others have focused more on training medical students to better address this and to increase their tolerance of uncertainty through concerted efforts [15]. These interventions, particularly if they took place early in medical education, could help to increase physicians’ awareness of the impact of uncertainty intolerance, and may also allow them to be more mindful of their own intolerance for ambiguity. The findings of this study emphasize the importance of NFCC in clinical practice and indicate that NFCC among practitioners may actually increase information gathering in some cases. NFCC may not, as was anticipated, hinder clinical practice, but may act as an incentive for physicians to locate answers for patients’ medical concerns.

## Conclusions

Future studies may do more to determine whether NFCC varies between clinical specialties. It is possible that certain specialties may actually benefit from high NFCC. In making rapid decisions, for instance, a physician’s ability to provide immediate and urgent care based on pattern-recognition may saves lives and treatment could be negatively impacted by increased thoughtfulness or a more planful approach. In other circumstances such as in complex cases, however, conscious decision making may benefit the decisions of expert doctors [16]. Further research would also help to understand whether the effects seen in this study actually impact clinical outcomes by measuring physician performance in an in vivo setting.

Given that our findings indicate decreased screening for possible treatable conditions or life circumstances, an important intervention may be increasing reminders for medical screening and improved standardization of screening practices in preventive care visits. Some studies have examined the impact of electronic health technology in reducing physician error and creating a greater sense of certainty in treating patients [17]. Electronic medical records may play an important role in this process as they allow for easier access to records and can be programed to remind physicians to ask certain screening questions [18]. This has been shown to be effective in increasing screening questions in other areas [19] and may help to reduce the impact of high NFCC on WWEs.

These results indicate that NFCC in ob/gyns may have an important impact on practice. This seems to be particularly true in terms of information gathering during WWEs. It is possible that increased training during residency or medical school could counteract the detrimental effects of NFCC, and may reduce it overall among clinicians [10]. Additionally, steps can be taken, particularly through increased use of electronic reminder systems, to orient physicians to the appropriate questions to ask patients.

## Additional file

Additional file 1:
**Decision-Making Under Uncertainty.**
