# Supplementary material for: Need to know: the need for cognitive closure impacts the clinical practice of obstetrician/gynecologists
Source: BMC Med Inform Decis Mak. 2014 Dec 24;14:122. doi: 10.1186/s12911-014-0122-6 (PMC4297425; doi:10.1186/s12911-014-0122-6)
Supplement: Additional file 1: — Decision-Making Under Uncertainty. [file 12911_2014_122_MOESM1_ESM.docx]

Additional File 1.

**Decision-Making Under Uncertainty:**

1. How often do you use each of the following when making the decision to prescribe a new drug?

|  | Almost Never | Rarely | Some-times | Often | Almost Always |
| --- | --- | --- | --- | --- | --- |
| Pharmaceutical brochures: | ➀ | ➁ | ➂ | ➃ | ➄ |
| Pharmaceutical reps: | ➀ | ➁ | ➂ | ➃ | ➄ |
| Other Advertisements: | ➀ | ➁ | ➂ | ➃ | ➄ |
| Consult with colleagues: | ➀ | ➁ | ➂ | ➃ | ➄ |
| Internet search: | ➀ | ➁ | ➂ | ➃ | ➄ |
| Journal articles: | ➀ | ➁ | ➂ | ➃ | ➄ |
| Educational seminars, meetings, or CME activities (not sponsored by pharmaceutical companies) | ➀ | ➁ | ➂ | ➃ | ➄ |
| Physicians’ Desk Reference (PDR) | ➀ | ➁ | ➂ | ➃ | ➄ |
| Pharmacist | ➀ | ➁ | ➂ | ➃ | ➄ |
| ACOG publications | ➀ | ➁ | ➂ | ➃ | ➄ |
| Food and Drug Administration (FDA) | ➀ | ➁ | ➂ | ➃ | ➄ |
| Other (specify): _________________ | ➀ | ➁ | ➂ | ➃ | ➄ |

1. How frequently did you consult with the following in the previous **month**?

|  | 0 times | 1 or 2 times | 3 or 4 times | 5 to 10 times | ≥10 times | N/A – this is my specialty; OR I do not see pregnant patients |
| --- | --- | --- | --- | --- | --- | --- |
| Genetic counselor | ➀ | ➁ | ➂ | ➃ | ➄ | ➅ |
| MFM specialist | ➀ | ➁ | ➂ | ➃ | ➄ | ➅ |
| Gynecological oncologist | ➀ | ➁ | ➂ | ➃ | ➄ | ➅ |
| Reproductive endocrinologist | ➀ | ➁ | ➂ | ➃ | ➄ | ➅ |
| Internist | ➀ | ➁ | ➂ | ➃ | ➄ | ➅ |
| Neonatologist | ➀ | ➁ | ➂ | ➃ | ➄ | ➅ |
| Other (specify): ________________________ | ➀ | ➁ | ➂ | ➃ | ➄ | ➅ |

1. Indicate the treatment(s) you are most likely to recommend for **non-pregnant** patients presenting with each of the following disorders. *(If you do not care for non-pregnant patients, please check here* ❒ *and skip to Question 4.)*

(*Please check your most likely course of action.* ***You may select more than one*** *if you typically provide more than one intervention for the listed concern.)* (OTC= over-the-counter).

|  | **Prescription Medication** | **Specialist Referral** | **You Counsel** | **Other (e.g. OTC, etc.)**  **(Please specify)** |
| --- | --- | --- | --- | --- |
| Urinary Tract Infection | ➀ | ➁ | ➂ | ➃ |
| Asthma | ➀ | ➁ | ➂ | ➃ |
| Diabetes | ➀ | ➁ | ➂ | ➃ |
| Hypertension | ➀ | ➁ | ➂ | ➃ |
| Frequent/Severe Headaches | ➀ | ➁ | ➂ | ➃ |
| Flu | ➀ | ➁ | ➂ | ➃ |
| Chronic anxiety | ➀ | ➁ | ➂ | ➃ |
| Chronic depressed mood | ➀ | ➁ | ➂ | ➃ |
| Chronic insomnia | ➀ | ➁ | ➂ | ➃ |

1. Rate how often you address the following with patients who come in for a periodic non-pregnant well-woman exam.

*(If you do not perform well-woman exams, please check here* ❒ *and skip to Question 5.)*

|  | **Never** | **Rarely** | **Sometimes** | **Often** | **Always** |
| --- | --- | --- | --- | --- | --- |
| Folic acid supplements (for women of reproductive age) | ➀ | ➁ | ➂ | ➃ | ➄ |
| Obesity (if overweight) | ➀ | ➁ | ➂ | ➃ | ➄ |
| Exercise | ➀ | ➁ | ➂ | ➃ | ➄ |
| Alcohol consumption | ➀ | ➁ | ➂ | ➃ | ➄ |
| Cigarette smoking | ➀ | ➁ | ➂ | ➃ | ➄ |
| Illegal drug use | ➀ | ➁ | ➂ | ➃ | ➄ |
| Prescription drug use | ➀ | ➁ | ➂ | ➃ | ➄ |
| Over the counter drug use | ➀ | ➁ | ➂ | ➃ | ➄ |
| Environmental toxins (work, hobbies, etc.) | ➀ | ➁ | ➂ | ➃ | ➄ |
| Family health history (heritable disorders) | ➀ | ➁ | ➂ | ➃ | ➄ |
| Sexual abuse | ➀ | ➁ | ➂ | ➃ | ➄ |
| Domestic Violence | ➀ | ➁ | ➂ | ➃ | ➄ |
| Mental Health (depression, anxiety, etc.) | ➀ | ➁ | ➂ | ➃ | ➄ |
| Caffeine Use | ➀ | ➁ | ➂ | ➃ | ➄ |

1. How likely are you to offer a TOLAC/VBAC to a patient with 1 prior low transverse c-section under the following circumstances:
   *(If you do not perform TOLAC/VBACs*, check here ❒ and skip to Question 9, the “**Periviable deliveries”** section.)
   *(If you do not perform obstetrics*, check here ❒ and skip to Question 29, the “**Work Questions”** section.)

|  | **Never** | **Rarely** | **Sometimes** | **Often** | **Always** |
| --- | --- | --- | --- | --- | --- |
| No prior vaginal deliveries | ➀ | ➁ | ➂ | ➃ | ➄ |
| One or more prior vaginal deliveries | ➀ | ➁ | ➂ | ➃ | ➄ |
| One or more prior successful VBACs | ➀ | ➁ | ➂ | ➃ | ➄ |
| Spontaneous labor | ➀ | ➁ | ➂ | ➃ | ➄ |
| Induction of labor | ➀ | ➁ | ➂ | ➃ | ➄ |
| Prior arrest of dilation or arrest of descent | ➀ | ➁ | ➂ | ➃ | ➄ |
| Patient desires TOLAC/VBAC despite having a low likelihood of success | ➀ | ➁ | ➂ | ➃ | ➄ |

1. Rate your degree of agreement or disagreement with the following statement:
   *I would favor limiting reimbursement for expensive drugs and procedures if that would help expand access to basic health care for those currently lacking such care.*

➀ Strongly Disagree ② Moderately Disagree ③ Moderately Agree ④ Strongly Agree

1. Indicate the degree to which you object (if at all), for moral reasons, to the following medical practice:
   *Using cost-effectiveness data to determine which treatments will be offered to patients.*

➀ No moral objection ② Moderate moral objection ③ Strong moral objection

1. INSTRUCTIONS: Read each of the following statements and decide how much you agree with each according to your beliefs and experiences *IN YOUR EVERYDAY LIFE*. Please respond according to the following scale.

|  | **strongly disagree** | **moderately disagree** | **slightly disagree** | **slightly agree** | **moderately agree** | **strongly agree** |
| --- | --- | --- | --- | --- | --- | --- |
| In cases of uncertainty, I prefer to make an immediate decision, whatever it may be. | ➀ | ➁ | ➂ | ➃ | ➄ | ➅ |
| When I find myself facing various, potentially valid, alternatives, I decide in favor of one of them quickly and without hesitation. | ➀ | ➁ | ➂ | ➃ | ➄ | ➅ |
| I have never been late for work or for an appointment. | ➀ | ➁ | ➂ | ➃ | ➄ | ➅ |
| I prefer to decide on the first available solution rather than to ponder at length what decision I should make. | ➀ | ➁ | ➂ | ➃ | ➄ | ➅ |
| I get very upset when things around me aren’t in their place. | ➀ | ➁ | ➂ | ➃ | ➄ | ➅ |
| Generally, I avoid participating in discussions on ambiguous and controversial problems. | ➀ | ➁ | ➂ | ➃ | ➄ | ➅ |
| When I need to confront a problem, I do not think about it too much and I decide without hesitation. | ➀ | ➁ | ➂ | ➃ | ➄ | ➅ |
| When I need to solve a problem, I generally do not waste time in considering diverse points of view about it. | ➀ | ➁ | ➂ | ➃ | ➄ | ➅ |
| I prefer to be with people who have the same ideas and tastes as myself. | ➀ | ➁ | ➂ | ➃ | ➄ | ➅ |
|  | **strongly disagree** | **moderately disagree** | **slightly disagree** | **slightly agree** | **moderately agree** | **strongly agree** |
| Generally, I do not search for alternative solutions to problems for which I already have a solution available. | ➀ | ➁ | ➂ | ➃ | ➄ | ➅ |
| I feel uncomfortable when I do not manage to give a quick response to problems that I face. | ➀ | ➁ | ➂ | ➃ | ➄ | ➅ |
| I have never hurt another person’s feelings. | ➀ | ➁ | ➂ | ➃ | ➄ | ➅ |
| Any solution to a problem is better than remaining in a state of uncertainty. | ➀ | ➁ | ➂ | ➃ | ➄ | ➅ |
| I prefer activities where it is always clear what is to be done and how it needs to be done. | ➀ | ➁ | ➂ | ➃ | ➄ | ➅ |
| After having found a solution to a problem, I believe that it is a useless waste of time to take into account diverse possible solutions. | ➀ | ➁ | ➂ | ➃ | ➄ | ➅ |
| I prefer things to which I am used to versus those I do not know, and cannot predict. | ➀ | ➁ | ➂ | ➃ | ➄ | ➅ |

**Work Questions**

1. Do you consider yourself: ➀ Mostly primary care provider ➁ Mostly specialist ➂ Both primary care provider and specialist
2. Approximately how many hours do you work each week? _____hrs
3. Regarding the amount of time you spend with your patients, would you like:
   ➀ MORE time with patients ➁ LESS time with patients ➂ Same amount of time with patients
4. In general, do you feel you have enough time to accomplish everything you want at work? ➀ Yes ➁ No ➂ It varies
5. Do you feel stressed *at work*? ➀ Not at all ➁ A little ➂ Somewhat ④ Very
6. How often do you read ACOG Practice Bulletins?
   ➀ Never ➁ Rarely ➂ Sometimes ④ Often ⑤ Always
7. Have you ever been named in a malpractice lawsuit that resulted in a settlement or judgment against you? ➀ No ➁ Yes
8. Do you supervise residents in your practice? ➀ No ➁ Yes
9. Are Trials of Labor or Vaginal Births After Cesarean Section (*TOLAC/VBACs)* permitted at your institution? ➀ No ➁ Yes

**Demographics:**

1. Your age: ________ 2. Gender: ➀ Male ➁ Female 3. Years in practice post residency: _______
2. State where primary practice is located:___________________
3. Please check your primary medical specialty (*check* ***one***)

➀ General Obstetrics and Gynecology ➁ Gynecology only ➂ Obstetrics only ④ Gynecologic Oncology
⑤ Reproductive Endocrinology/Infertility ⑥ Maternal/Fetal Medicine ⑦ Urogynecology ⑧ Other (specify)______

1. With which race/ethnicity do you identify? *(check* ***all*** *that apply)*

➀ White/Caucasian ➁ Black/African American ➂ Hispanic/Latin American

④ Asian ⑤ Native Hawaiian/Pacific Islander ⑥ American Indian/Alaskan Native

⑦ Biracial/Multiracial ⑧ Other ________________________

1. Which best describes your current practice?

➀ Solo Practice ➁ OB/GYN partnership/group ➂ Multi-specialty group

④ HMO (staff model) ⑤ University full-time faculty and practice ⑥ Other __________________

1. Which of the following do you consider your primary medical specialty? (*check* ***one***)

➀ General ob-gyn ➁ Gynecology only ➂ Obstetrics only

④ Maternal Fetal Medicine ⑤ Gynecologic oncology ⑥ Reproductive endocrinology

⑦ Urogynecology ⑧ Other__________________

1. Which of the following best describes the location of your practice?

➀ Urban, inner city ➁ Urban, non-inner city ➂ Suburban

④ Town of 5,000-50,000 ⑤ Rural, or town of 5,000 or less ⑥ Other __________________

*The following questions are optional; please only respond if you feel comfortable:*

1. What is your religious affiliation?

➀ Christian – Mainline Protestant ➁ Christian – Evangelical Protestant ➂ Christian –Catholic

④ Muslim ⑤ Jewish ⑥ Buddhist

⑦ Hindu ⑧ Other ____________ ⑨ None

1. How often do you attend religious services?

➀ Never ➁ Less than once a year ➂ About 1-2 times/year

④ Several times/year ⑤ About 1/month ⑥ 2-3 times/month

⑦ Nearly every week ⑧ Every week ⑨ Several times/week

1. How important would you say your religion is in your own life?

➀ The most important ➁ Very important ➂ Fairly important

④ Not very important ⑤ Not applicable/I have no religion

1. Regarding social issues (politically), do you consider yourself: ➀ Liberal ➁ Moderate ➂ Conservative ④ Other
2. Are you a parent ➀ No ➁ Yes 🡪 If yes, do any of your children have special needs? ① No ➁ Yes
